# Risk factors for delayed gastric emptying following laparoscopic repair of very large hiatus hernias

**DOI:** 10.1002/bjs5.11

**Published:** 2017-08-28

**Authors:** C. Tog, D. S. Liu, H. K. Lim, P. Stiven, S. K. Thompson, D. I. Watson, A. Aly

**Affiliations:** ^1^ Department of Surgery Austin Hospital Heidelberg Victoria Australia; ^2^ Division of Cancer Surgery Peter MacCallum Cancer Centre Melbourne Victoria Australia; ^3^ University of Adelaide Discipline of Surgery Royal Adelaide Hospital Adelaide South Australia Australia; ^4^ Flinders University Department of Surgery Flinders Medical Centre Bedford Park South Australia Australia

## Abstract

**Background:**

Delayed gastric emptying can complicate surgery for hiatus hernia. The aim of this study was to quantify its incidence following laparoscopic repair of very large hiatus hernias, identify key risk factors for its occurrence and determine its impact on clinical outcomes.

**Methods:**

Data collected from a randomized trial of patients who underwent laparoscopic mesh versus sutured repair of very large hiatus hernias (more than 50 per cent of stomach in chest) were analysed retrospectively. Delayed gastric emptying was defined as endoscopic evidence of solid food in the stomach after fasting for 6 h at 6 months after surgery.

**Results:**

Delayed gastric emptying occurred in 19 of 102 patients (18·6 per cent). In univariable analysis, type 2 paraoesophageal hernia (relative risk (RR) 3·15, 95 per cent c.i. 1·41 to 7·06), concurrent anterior and posterior hiatal repair (RR 2·66, 1·14 to 6·18), hernia sac excision (RR 4·85, 1·65 to 14·24), 270°/360° fundoplication (RR 3·64, 1·72 to 7·68), division of short gastric vessels (RR 6·82, 2·12 to 21·90) and revisional surgery (RR 3·69, 1·73 to 7·87) correlated with delayed gastric emptying. In multivariable analysis, division of short gastric vessels (RR 6·27, 1·85 to 21·26) and revisional surgery (RR 6·19, 1·32 to 28·96) were independently associated with delayed gastric emptying. Delayed gastric emptying correlated with adverse gastrointestinal symptomatology, including higher rates of bloating, nausea, vomiting and anorexia, as well as reduced patient satisfaction with the operation and recovery.

**Conclusion:**

Delayed gastric emptying following large hiatus hernia repair is common and associated with adverse symptoms and reduced patient satisfaction. Division of short gastric vessels and revisional surgery were independently associated with its occurrence.

## Introduction

Since its innovation in the 1990s^1^, ^2^, ^3^, laparoscopic surgery for the treatment of hiatus hernia has become standard practice. With continued improvement in laparoscopic technology and techniques, this approach has been increasingly applied to repair very large hiatus hernias^4^ ^5^. Despite variable rates of hernia recurrence^5^, ^6^, ^7^, the outcomes of surgery are generally encouraging, with substantial symptomatic relief of reflux and low associated operative mortality or morbidity^8^ ^9^. Most follow‐up studies have focused on minimizing hernia recurrence and its associated symptomatology^6^ ^7^. In contrast, little is known about delayed gastric emptying following laparoscopic repair of very large hiatus hernias.

Delayed gastric emptying implies prolonged retention of food in the stomach and is usually diagnosed by gastroscopy, barium meal or isotope gastric‐emptying studies. There is no consensus definition across these tests^10^. Nuclear scintigraphy has been the test used most commonly^11^, although a recent study^12^ suggested that endoscopic evidence of food retention following routine fasting closely correlates with severe delayed gastric emptying on nuclear scintigraphy.

Antireflux surgery is generally thought to facilitate gastric motility^13^, ^14^, ^15^, ^16^; however, a proportion of patients develop delayed gastric emptying, which may negate the benefits of surgery^8^ ^9^, ^17^. Although the pathophysiology of postoperative delayed gastric emptying is likely to be multifactorial, vagal nerve injury may be an important contributor^18^, ^19^, ^20^. This in turn may relate to the extent of surgical dissection near the hiatus^17^. Despite a relatively low rate of delayed gastric emptying reported for repair of small hiatus hernias^7^, ^8^, ^9^, its incidence and predisposing factors after surgery for very large hiatus hernias remain unknown. This study sought to identify the incidence of delayed gastric emptying following surgery for very large hiatus hernias, highlight risk factors for its occurrence and examine its medium‐term impact on patients, based on data collected from a prospective randomized trial.

## Methods

Data from an RCT of patients undergoing laparoscopic repair of very large hiatus hernias were analysed. The trial protocol and outcomes have been reported previously^5^. Briefly, this trial randomized 126 patients to sutured *versus* mesh repair of very large hiatus hernias (more than 50 per cent of stomach contained in the thoracic cavity as defined by endoscopy, CT and/or barium X‐ray), performed by nine specialist surgeons across three university hospitals and one private centre in Australia, between February 2006 and September 2012. This study and the original randomized trial were approved by the human research ethics committee at each participating hospital, and conducted in accordance with the National Health and Medical Research Council (NHMRC) of Australia's guidelines on human experimentation.

### Surgical technique

The laparoscopic approach to repair large hiatus hernias was standardized across the four centres^5^. Surgery comprised full dissection and removal of the hernia sac from the mediastinum, and full reduction of the contents into the abdomen. Subsequent excision of the hernia sac from the gastric cardia was at the operating surgeon's discretion. No oesophageal lengthening procedures were undertaken. The oesophageal hiatus was narrowed using posterior sutures in all patients, with additional anterior hiatal sutures placed if the surgeon considered these necessary to minimize tension on the sutured repair. For patients who were randomized to receive mesh reinforcement, a rectangular (2–3 × 4–5 cm) piece of mesh, either 4‐ply Surgisis^®^ ES (Cook Biotech, West Lafayette, Indiana, USA) or TiMESH^®^ (PFM Medical, Köln, Germany), was placed in an onlay fashion over the posterior hiatal repair sutures and the hiatal pillars without encircling the oesophagus. The mesh was secured using either sutures, glue or a mechanical ‘tacker’ (ProTack™; Covidien, New Haven, Connecticut, USA). A fundoplication procedure was added in all patients. The extent of fundoplication and use of an oesophageal bougie, as well as the decision to divide the short gastric vessels, were at the discretion of the operating surgeon.

### Study endpoints and definitions

The main endpoint of this observational study was the incidence of delayed gastric emptying following repair of large hiatus hernias. Other endpoints included clinical symptoms as well as patient satisfaction with their surgery and postoperative recovery. Delayed gastric emptying was defined by the presence of solid food residue in the stomach seen at gastroscopy performed 6 months after surgery.

Routine gastroscopic examinations were performed as part of the trial protocol to assess the integrity of the hiatal repair. The presence of food residue in the stomach was documented routinely by the endoscopist. All patients were fasted for at least 6 h before gastroscopy.

Follow‐up of clinical symptoms and patient satisfaction was undertaken by research nurses using a structured questionnaire, applied 3, 6 and 12 months after surgery, as described previously^5^ ^21^. For the purpose of this study, five main symptoms relevant to gastroparesis were included (incidence of epigastric pain, postprandial bloating, anorexia, nausea and vomiting), and changes in the patient's weight following surgery were recorded. Overall satisfaction with the outcome of surgery was assessed using a 0–10 analogue scale, with 0 and 10 indicating highly dissatisfied and satisfied respectively. Additionally, during each follow‐up visit, patients were asked if they believed their original decision to undergo surgery was correct. All endoscopists, patients and research nurses were blinded to the operation variant in the original trial.

### Data collection

Data were extracted from a prospectively developed database on to a standard pro forma. This included patient demographics, body mass index, the presence of diabetes, medications, hernia type and size, operative variables (use of a bougie, number of crural sutures placed, crural repair location, application of mesh, method of mesh anchorage, extent of fundoplication, excision of hernia sac, division of short gastric vessels, number of intraoperative complications, duration of surgery and its difficulty), length of hospital stay, reherniation rates, and the need for revisional surgery owing to postoperative complications (such as reherniation and dysphagia).

### Statistical analysis

Cohorts of patients who did, or did not develop postoperative delayed gastric emptying were compared. Categorical variables were analysed with Fisher's exact test. Unpaired Student's *t* test and Mann–Whitney *U* test were used to analyse parametric and non‐parametric data respectively. To determine independent predictors of postoperative delayed gastric emptying, a multivariable analysis using a forward stepwise regression algorithm was subsequently performed. This was based on parameters found in univariable analysis with *P* ≤ 0·050. A two‐tailed *P* ≤ 0·050 and a 95 per cent c.i. around the relative risk (RR) that did not include 1·00 was considered statistically significant. Statistical analyses were conducted using SPSS^®^ version 22.0 (IBM, Armonk, New York, USA) and Prism^®^ version 6.0 (GraphPad, San Diego, California, USA).

## Results

### Incidence of delayed gastric emptying after laparoscopic repair of very large hiatus hernias

Of 126 patients who had laparoscopic repair of a very large hiatus hernia, 102 underwent postoperative gastroscopy and were included in this study. The remaining 24 patients were either lost to follow‐up or did not have gastroscopy at 6 months after surgery, and were excluded. The incidence of delayed gastric emptying, as defined by gastroscopy at 6 months, was 18·6 per cent (19 of 102 patients). The demographic characteristics of these patients are described in *Table* 1.

**Table 1:** Patient characteristics

	DGE (*n* = 19)	No DGE (*n* = 83)	*P* ¶	Relative risk†
Age (years)*	67 (47–85)	68 (43–88)	0·751#	–
Sex				
M	6 (32)	26 (31)	1·000	1·01 (0·35, 2·96)
F	13 (68)	57 (69)		1·00 (reference)
Body mass index (kg/m^2^)*	27·8 (21·1–32·6)	28·4 (21·2–54·7)	0·558#	–
Diabetes				
Yes	2 (11)	13 (16)	0·731	0·68 (0·18, 2·66)
No	13 (68)	51 (61)		1·00 (reference)
Unknown	4 (21)	19 (23)		1·00 (reference)
PPI use				
Yes	13 (68)	58 (70)	1·000	0·94 (0·40, 2·26)
No	6 (32)	25 (30)		1·00 (reference)
Prokinetic use‡				
Yes	1 (5)	1 (1)	0·339	2·78 (0·65, 11·82)
No	18 (95)	82 (99)		1·00 (reference)
Antikinetic use§				
Yes	3 (16)	5 (6)	0·166	2·20 (0·81, 5·99)
No	16 (84)	78 (94)		1·00 (reference)
Hospital				
1	3 (16)	24 (29)	0·387	0·52 (0·16, 1·65)
2	7 (37)	46 (55)		1·00 (reference)
3	7 (37)	12 (14)		1·00 (reference)
4	2 (11)	1 (1)		1·00 (reference)

Values in parentheses are percentages unless indicated otherwise; values are

* median (range) and

† 95 per cent confidence intervals.

‡ Prokinetics included metoclopramide and domperidone;

§ antikinetics included loperamide, opioids and antidepressants.

GDE, delayed gastric emptying; PPI, proton pump inhibitor.

¶ Fisher's exact test, except

# Mann–Whitney *U* test.

### Factors associated with delayed gastric emptying

Comparisons of demographic and operative characteristics between patients who did and those who did not develop delayed gastric emptying indicated that: the presence of type 2 (rolling) paraoesophageal hernia (RR 3·15, 95 per cent c.i. 1·41 to 7·06), placement of both anterior and posterior hiatal sutures (RR 2·66, 1·14 to 6·18), excision of hernia sac from the gastric cardia (RR 4·85, 1·65 to 14·24), division of short gastric blood vessels (RR 6·82, 2·12 to 21·90), performance of a 270°/360° fundoplication (RR 3·64, 1·72 to 7·68) and revisional surgery (RR 3·69, 1·73 to 7·87) for either acute reherniation (delayed gastric emptying group, 4; control group, 2) or redo fundoplication (delayed gastric emptying group, 1; control group, 2) were significant risk factors for delayed gastric emptying on univariable analysis (*Table* 2). All reoperations were conducted within 1 week of the initial operation. Patients who developed delayed gastric emptying had a significantly longer hospital stay after their initial procedure (median 5 days *versus* 3 days in the control group; *P* = 0·001).

**Table 2:** Surgical factors and their association with postoperative delayed gastric emptying

	DGE (*n* = 19)	No DGE (*n* = 83)	*P* ‡	Relative risk†
Hernia type				
Rolling	11 (58)	20 (24)	0·006	3·15 (1·41, 7·06)
Sliding	1 (5)	13 (16)		1·00 (reference)
Mixed	7 (37)	50 (60)		1·00 (reference)
% stomach in hernia sac				
100	4 (21)	11 (13)	0·437	1·55 (0·59, 4·03)
75–99	7 (37)	24 (29)		1·00 (reference)
50–74	8 (42)	48 (58)		1·00 (reference)
Bougie use				
Yes	14 (74)	51 (61)	0·430	1·76 (0·58, 5·35)
No	5 (26)	32 (39)		1·00 (reference)
No. of hiatus repair sutures*	5 (2–12)	5 (2–10)	0·664‡	–
Hiatus repair location				
Anterior and posterior	12 (63)	28 (34)	0·035	2·66 (1·14, 6·18)
Posterior	7 (37)	55 (66)		1·00 (reference)
Mesh use				
Yes	11 (58)	55 (66)	0·596	0·70 (0·25, 1·94)
No	8 (42)	28 (34)		1·00 (reference)
Mesh anchorage				
Tacker	9 (82)	50 (91)	0·330	0·53 (0·14, 1·99)
Glue	2 (18)	3 (5)		1·00 (reference)
Sutures	0 (0)	2 (4)		1·00 (reference)
Sac excision				
Yes	9 (47)	13 (16)	0·002	4·85 (1·65, 14·24)
No	10 (53)	70 (84)		1·00 (reference)
Short gastric vessels				
Divided	8 (42)	8 (10)	0·002	6·82 (2·12, 21·90)
Preserved	11 (58)	75 (90)		1·00 (reference)
Fundoplication type				
360° Nissen	1 (5)	1 (1)		
			0·003	3·64 (1·72, 7·68)
270°Toupet	7 (37)	8 (10)		
180° anterior	7 (37)	50 (60)		
				1·00 (reference)
90° anterior	4 (21)	24 (29)		
Intraoperative complications				
Yes	6 (32)	16 (19)	0·352	1·68 (0·72, 3·91)
No	13 (68)	67 (81)		1·00 (reference)
Duration of surgery (min)*	112·5 (35–200)	107·5 (45–390)	0·731§	–
Operation difficulty score*	6 (2–9)	5 (2–10)	0·102§	–
Acute revisional surgery				
Yes	5 (26)	4 (5)	0·011	3·69 (1·73, 7·87)
No	14 (74)	79 (95)		1·00 (reference)
Postoperative complications				
Yes	7 (37)	15 (18)	0·118	2·64 (0·89, 7·84)
No	12 (63)	68 (82)		1·00 (reference)
Duration of hospital stay (days)*	5 (2–30)	3 (1–43)	0·001§	–
Reherniation at 1 year				
Yes	3 (16)	34 (41)	0·062	1·22 (1·01, 1·46)
No	16 (84)	49 (59)		1·00 (reference)

Values in parentheses are percentages unless indicated otherwise; values are

* median (range) and

† 95 per cent confidence intervals.

DGE, delayed gastric emptying.

‡ Fisher's exact test, except

§ Mann–Whitney *U* test.

Based on multivariable analysis, division of the short gastric vessels (RR 6·27, 95 per cent c.i. 1·85 to 21·26; *P* = 0·003) and acute revisional surgery (RR 6·19, 1·32 to 28·96; *P* = 0·021) independently predicted the development of delayed gastric emptying at 6 months after laparoscopic repair of very large hiatus hernias (*Table* 3).

**Table 3:** Multivariable logistic regression analysis of postoperative delayed gastric emptying

	Relative risk	*P*
Short gastric vessels		
Division	6·27 (1·85, 21·26)	0·003
Preserved	1·00 (reference)	
Acute revision surgery		
Yes	6·19 (1·32, 28·96)	0·021
No	1·00 (reference)	
Hernia type		
Mixed	2·08 (0·20, 21·48)	0·54
Rolling	4·25 (0·42, 42·80)	0·22
Sliding	1·00 (reference)	
Hiatus repair location		
Anterior and posterior	2·44 (0·71, 8·41)	0·158
Posterior	1·00 (reference)	
Sac excision		
No	0·21 (0·01, 5·97)	0·363
Yes	1·00 (reference)	
Fundoplication type		
360° Nissen	2·87 (0·09, 91·54)	0·551
270° Toupet	2·61 (0·17, 40·42)	0·494
180° anterior	0·72 (0·17, 3·06)	0·660
90° anterior	1·00 (reference)	

Values in parentheses are 95 per cent confidence intervals.

### Impact of delayed gastric emptying on patient outcomes

**Figure 1:**
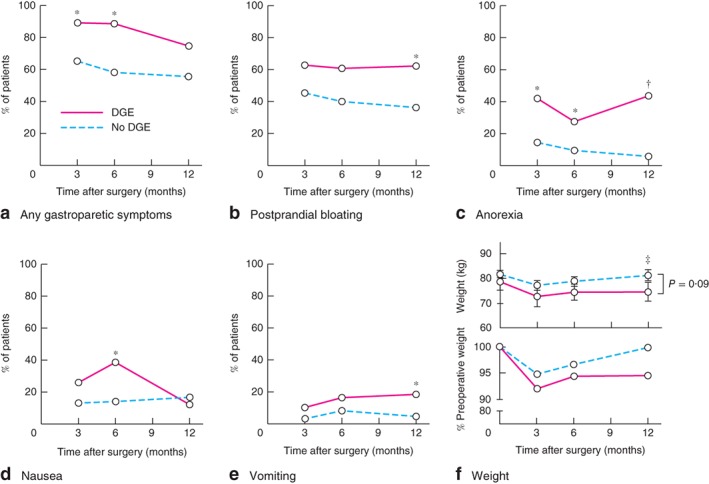
Impact of postoperative delayed gastric emptying (DGE) on patients' weight and symptoms. **a** Overall gastroparetic symptoms, **b** postprandial bloating, **c** anorexia, **d** nausea and **e** vomiting in patients with and those without DGE, assessed at 3, 6 and 12 months after hiatus hernia repair. **f** Changes in bodyweight (top: mean(s.e.m.) absolute bodyweight; bottom: percentage of preoperative bodyweight) over the same period. *P < 0·050, †P < 0·001, ‡P = 0·09 (**a–e** Fisher's exact test; **f** unpaired Student's t test)

**Figure 2:**
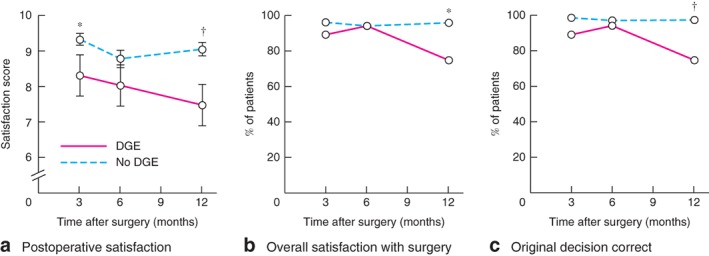
Impact of postoperative delayed gastric emptying (DGE) on patient satisfaction with surgery. **a** Mean(s.e.m.) satisfaction score (0, least satisfied; 10, most satisfied), **b** overall satisfaction with surgery and **c** belief that initial decision to undergo surgery was correct in patients with and those without DGE, assessed at 3, 6 and 12 months after hiatus hernia repair. *P < 0·050, †P < 0·010 (**a** unpaired Student's t test; **b,c** Fisher's exact test)

Patients who developed delayed gastric emptying experienced significantly more symptoms relating to gastroparesis, including postprandial bloating, anorexia, nausea and vomiting episodes, than patients who did not have delayed gastric emptying (*Fig*. 1 *a–e*). Many of these symptoms arose soon after surgery and persisted at 1‐year follow‐up. Consistently, patients who had delayed gastric emptying lost more weight after surgery and experienced difficulty returning to their baseline bodyweight (*Fig*. 1 *f*). These patients were also more dissatisfied with their surgery and postoperative recovery (*Fig*. 2 *a,b*). They were more likely to regret their initial decision for surgery than those who did not develop delayed gastric emptying (*Fig*. 2 *c*).

## Discussion

Delayed gastric emptying following laparoscopic repair of very large hiatus hernias occurred in almost one in five patients. From the patient's perspective this was important. Those with delayed gastric emptying experienced significantly worse gastrointestinal symptoms and side‐effects. As a consequence, they were more likely to be dissatisfied with their postoperative recovery and initial choice for surgery. Multiple surgical risk factors for delayed gastric emptying were identified. Division of short gastric vessels, and redo fundoplication or revisional hiatus hernia surgery were independent predictors of postoperative delayed gastric emptying. Interestingly, none of the demographic characteristics was found to be significantly associated with delayed gastric emptying in the present cohort. Although diabetes is a recognized risk factor for gastroparesis, this is restricted mainly to patients with poorly controlled insulin‐dependent diabetes^10^. In the present study, only one of 15 patients with diabetes was insulin‐dependent, and most had adequate glycaemic control.

The reported rate of delayed gastric emptying following primary laparoscopic repair of paraoesophageal hernias of any size is 0–3 per cent^7^, ^8^, ^9^. This rate increases with the number of revisional surgery procedures to as high as 75 per cent^17^. It is difficult, however, to compare directly the results from these studies with those of the present study. First, there is a discrepancy in hernia size between studies. Previous reports included patients with paraoesophageal hernias of any size, with most being relatively small. Given that large hiatus hernias are technically more demanding to repair due to increased adhesions and anatomical distortions, which may predispose to postoperative gastroparesis^17^, greater hernia size may thus partly account for the higher rate of delayed gastric emptying observed in the present study. Second, the definition of delayed gastric emptying also varies between published reports.

The finding in the present study that revisional surgery predisposed to delayed gastric emptying is concordant with an earlier report^17^ in which the rate of delayed gastric emptying increased from 12 per cent after first surgical revision to over 75 per cent following three revisions. Although no study has formally implicated division of short gastric vessels as a risk factor for delayed gastric empting, several randomized trials and meta‐analyses have found that routine division of short gastric vessels during laparoscopic fundoplication is associated with higher rates of postprandial bloating^22^, ^23^, ^24^, which may reflect delayed gastric emptying. Despite the rationale that division of short gastric vessels may facilitate the creation of a tension‐free fundoplication and minimize the risk of postoperative dysphagia^25^, multiple studies^26^, ^27^, ^28^, ^29^ have demonstrated that this intraoperative manoeuvre does not influence swallowing outcomes after antireflux surgery. Therefore, some surgeons no longer routinely divide the short gastric vessels^30^, ^31^, ^32^. The present study lends support to this practice.

The pathogenesis of postoperative delayed gastric emptying is unclear. One hypothesis is that this complication results from accidental intraoperative vagotomy^19^ ^33^. Owing to the close anatomical relationship between the vagal trunks and the distal oesophagus, gastro‐oesophageal junction and proximal stomach, it has been postulated that the combination of extensive sac dissection, oesophageal mobilization and gastric fundus manipulation predisposes to accidental vagotomy^15^ ^16^, ^33^ ^34^. This is further complicated by the observation that vagal nerve anatomy at the level of the hiatus and gastric fundus is highly variable, with studies^35^, ^36^, ^37^, ^38^ reporting accessory vagal trunks or fibres in over 40 per cent of patients. These accessory fibres typically arise from early division from the left and right vagal nerves, or directly from the oesophageal plexus. Many of these fibres travel left of the main trunks and innervate the fundus both anteriorly and posteriorly close to the sympathetic branches travelling in the gastrophrenic ligament^35^. Vagal nerve damage may lead to excessive relaxation of the fundus, hypomotility of the corpus and antrum, and desynchronization of gastric pacemaker activity, resulting in delayed gastric emptying^39^, ^40^, ^41^. In support of this hypothesis, the risk factors identified by univariable analysis in this study all share a common theme. They typically involve more surgical manipulation with high‐energy devices, in a relatively confined space in close proximity to the vagal trunks and its branches. Transmitted energy during the division of short gastric vessels may result in accidental fundal vagotomy.

Other mechanisms might contribute to delayed gastric emptying. This problem may result from chronic entrapment of the stomach inside the hernia sac^42^, ^43^, ^44^. Restorative surgery might exacerbate this or simply expose symptoms that were minor or absent before surgery compared with other symptoms attributable to these large hernias.

The limitations of this study are inherent in its design. No formal gastric emptying studies were conducted to validate the gastroscopy findings. The presence of delayed gastric emptying was not documented before hiatus hernia repair. The presence of a hiatus hernia, in any event, can make gastric emptying studies difficult to interpret, thus limiting their value in predicting postoperative delayed gastric emptying^17^. The aetiology of preoperative delayed gastric emptying may differ from that of postoperative delayed emptying; the former may reflect entrapment of a large portion of stomach within the thoracic cavity, whereas surgical manipulation probably contributes to the latter. Vagal function was not tested, and the absolute number of patients with delayed gastric emptying in the present study was small. Owing to uneven recruitment from the four participating centres, there may be centre‐specific bias in the rate of delayed gastric emptying.
